# Discussions of the Mississippi Valley Association

**Published:** 1857-03

**Authors:** 


### DISCUSSIONS OF THE MISSISSIPPI VALLEY ASSOCIATION.

### FIRST DAY—AFTERNOON SESSION.

*Question No. I.—Is Annealed Foil, as now used, preferable to Blocks for Filling, and does it Weld as perfectly as "Crystal Gold?"*

Dr. Bonsall said he annealed foil in one-eighth or one-tenth of his cases; he did not know much of crystal gold; it was not as satisfactory in his hands as in others, and therefore he used it little. His use of crystal gold would depend upon the shape of the cavity in a tooth; he would be fearful of annealing foil when the mouth of the cavity was small.

Dr. James Taylor remarked that foil becomes moist from the atmosphere; foil not in use unless carefully wrapped up, will become moist. Foil in a wet state cannot be made as compact as in a dry state. He annealed after manipulation. Foil that becomes hard he annealed; he was in the habit of sending for a single book of foil only at a time, as foil recently made is the best, and that lately annealed the most convenient for use. When he had a large amount of foil on hand, he had to anneal it often. When he used foil in blocks he annealed that they might regain that adhesiveness lost by exposure to moisture. He did not know if foil would adhere better than crystal gold, which he was not much in the habit of using. A good plug may be made of crystal gold, but he thought crystal gold apt to crumble. In using crystal gold and afterward in examining the teeth in which it had been used, he had found in the dentine a lamina of bone that looked as if it had been acted upon by muriatic acid. He had learned to manipulate foils, and he deemed that the best mode of plugging which had accomplished his purpose. He could not make as good plugs of crystal gold as of foil, and he thought he could plug a cavity better with foil than any one with crystal gold. He could do much of the labor with foil outside of the cavity, and this was with him very desirable. His object was to reduce the time requisite for the introduction of gold into the tooth as much as possible. The ease of the patient and the operator demands this. The brevity of time enables the operator to keep the cavity dry, upon which so much depends. In difficult cavities in using crystal gold, the crystals become moist, and where moisture does take place there is less injury to foil than to crystal gold. Foil does not crumble. He was disinclined to throw aside foil after laboring for a number of years to use it successfully. A plug that will not come out and will exclude moisture, he deemed a perfect filling, and such a plug can be made of annealed foil.

Dr. Stipher had been experimenting a little; had used foil altogether, and had found annealed foil the best. His instruments were better adapted to using foil.

Dr. BAXTER used foil. With foil he could fill a cavity more rapidly than with crystal gold, and therefore he preferred foil.

Dr. RICHARDSON had had no experience with annealed foil as recently used. He had been in the habit of filling with foil as blocks. The adhesiveness of foil was proved by the ring Dr. James Taylor had shown to the members of the Convention, which ring was made of foil, and had been worn for some time. In filling a tooth the excellence of the filling depended upon the care in preparing and putting in the gold. More skill is required in using crystal gold and annealed foil than foil in its ordinary state. He had filled with crystal gold, and he believed it good; he thought it would make excellent plugs.

DR. KEELY said, that while some liked the crystal gold, his experience had induced him to prefer foil well annealed.

DR. ULLERY used gold foil, sometimes annealed and sometimes not. He could make a better filling by annealing. He had known crystal gold to crumble, and had been compelled to take it out; although he believed that those operators had been successful with crystal gold, who had had much practice with it. He did not know how long crystal gold would last; thought it would last as long as foil. Annealed foil made, he said, good enough plugs for him.

DR. OSMOND said, that annealing foil made it hard. By annealing gold he made a good filling. Some time before he had a book of gold foil that had become rather damp. He could not use the foil, and therefore he put it in between two flat irons, heated to the temperature that laundresses give them; letting the steam escape by raising the iron occasionally. After undergoing this process, he found the foil dry and good; he wanted no better. He preferred this process of removing the moisture from foil, because placing it over a flame is apt to harden it too much.

Dr. Taft annealed foil by the method explained by Prof. Arthur; he either tore it up and then annealed it, or annealed it first. He liked annealed foil for filling, because of its cohesiveness, and its capacity to be solidified. There was no doubt of the cohesiveness of its particles, to such an extent that they could not be separated. In filling a cavity of a tooth, the annealed foil will sometimes harden in the centre and not at the outside. If an operator carelessly make his pellets too large, they are apt to become fastened at the centre, and do not go to the walls of the cavity. There is much difficulty in making an adaptation to all parts of the cavity. Annealed foil may be best used in small pellets, and in applying them to the walls of the cavity first. It is difficult to fill some cavities with foil. Operators in endeavoring to work the annealed foil over or under a projection of a tooth, fail to make a perfect adaptation. If one wish to use annealed foil, perfectly, it is objectionable to form it in blocks; it is better torn into fragments. He in general, however, preferred crystal gold.

---

SECOND DAY—MORNING SESSION.

Dr. McClelland had tried annealed foil, and found some difficulties in using it. In approximal cavities he had used foil. In filling cavities with annealed foil, he had discovered that on account of its cohesiveness, it would adhere to the plugging instrument when he wished to draw it out. He preferred ordinary foil perfectly dry to annealed foil. When foil was moist, he placed it on a platinum plate over a spirit lamp. He used crystal gold in certain cases, that seemed to require it.

Dr. Spalding knew something about the use of blocks and about crystal gold. He had not tried annealed foil as described by Prof. Arthur, but had used crystal gold extensively. A few days before, he had examined crystal gold plugs inserted by him some years previously, and had found them in excellent condition. He had rarely known crystal gold to crumble, and when it did, he attributed it to the impurity of the article. He always used crystal gold for apertures deep and narrow; for filling underneath projections and shelving portions of the teeth. If the cavity was not large enough, he would excavate it. Where the cavity was irregular, he rendered its walls perpendicular with crystal gold before using foil, which he usually formed into cylinders. The greater portion of his foil he annealed; but for finishing the plug he did not anneal. The adhesiveness of foil depends upon its purity. While the gold at the manufacturers is in the form of a precipitate, it is washed, and if washed clean, it will adhere. Great care should be taken to remove from the precipitated gold the iron, the sulphate of which is ordinarily used for the purpose of precipitation. The welding together of foil depends upon its mechanical position; the layers should be placed on one another, so that each particle may adhere to its fellow. In the form of pellets, foil requires great force and skill in driving them home. Foil in his hands, not only received moisture, but a portion of organic matter, and consequently before using it, he applied heat enough to expel the moisture and consume the organic matter. In what is termed annealed foil, he did not know that enough heat was applied to be considered annealing, properly speaking, which meant the application of heat sufficient to give the particles an opportunity to rearrange themselves. Foil, to be fit for use, must be clean and pure. In most cavities he used gold foil in the form of blocks or cylinders; he had used crystal gold also, as he had stated, but could not use it with the same facility as foil. He occasionally used pellets, but deemed them imperfect. His foil was No. 4, but considered the number unimportant.

Dr. Branch. With him crystal gold had proved a failure, he thought mercury was in the gold, from its turning black after it had been employed in filling. He had obtained all his knowledge of crystal gold from a treatise he had read on the subject. He had used unannealed gold for a long time; but about five years before, he began to anneal his foil, and had recently been employing adhesive foil in his practice. His custom was to fill from the bottom of a cavity up. Where there was no irregularity he made one. His operations had been most satisfactory with adhesive foil. Where an incisor was broken off, or where a cavity was shallow, he drilled one or two small holes into the teeth, and introduced adhesive foil therein. The instruments he used in plugging were sharp and swallow-tailed in shape, but at the close of the work, he employed blunt implements. He had known a very successful operator, Dr. Ballard, whose instruments,—small and star-shaped at the point,—packed the gold by interlocking. As to annealed foil he believes with Dr. Spalding. Foil was often better prepared for filling teeth by heating, the particles seemed better arranged and much more apt to firmly cohere.

Dr. Hamill, had during the past year used crystal gold for filling teeth, and preferred it. He thought when one had a good article, and persisted in using it, he would prefer it to foil, which, however, he employed, where rapidity was necessary.

Dr. Walker, had had no experience in crystal gold. The greatest recommendation of foil is, that it can be entirely prepared for filling outside of the mouth.

Dr. Baxter, remarked that gold foil by pressing it directly against other gold, would not adhere; that the adhesion arose not from the pressure, but the manner of pressure. An oblique method, therefore, of using instruments in plugging teeth, would be apt to render the filling most secure.

Dr. Taft, desired to call attention to three of the chief points evolved during the discussion. I. Dr. Spalding's method of filling under cavities with crystal gold. II. The filling of cavities referred to by the same gentleman, with annealed gold, and then closing with unannealed foil to prevent adhesion. III. The method, mentioned by Dr. Branch, of using drills for making retaining points, where a cavity was shallow, that the filling might be more secure. The latter point was introduced to the public, by Dr. A. S. Talbert, in a paper read here several years ago.

Dr. McClelland, had found the drilling of teeth, particularly front ones, from their sensitiveness, painful to patients, and therefore, preferred excavation. In such cases, he employed a hatchet. He first inserted a block extending outward, then a block against the gum. He used a blunt instrument first, and afterward a sharp one. After plugging he filled, and repeated the operation of filling at different times. For polishing fillings, he employed the leather that had been used by daguerreotypists, as it was full of crocus.

Dr. Spalding, in filling, used blocks of a tapering shape, and left the last points to be plugged of a conical form. In the last filling, he introduced instead of a cylinder, a cone with the apex downwards.

Dr. James Taylor, in double cavities, central and approximal, used cylinders for filling and a sharp instrument. The longer he practiced the more he adopted his cylinders and cones to the cavity. The perfection of filling depends upon its solidity. He was opposed to plugging front teeth in any way, that revealed the gold. In filling, he did not build up upon a slender foundation, but cut away, and built where there was strength; but he doubted if this was good practice.

Dr. Spalding folded the foil before filling, after cutting it in the shape of ribbons; but did not know if this was the best form. He obtained his gold from the manufacturers, five or six layers together, and cut it with tailors' shears. He thought the foil more adhesive in this way.

Mr. Leslie, manufacturer of foil in the city, being present, was invited to address the Convention, and show by experiment the adhesiveness of foil, and his mode of annealing it. He said that dentists were largely dependent upon the manufacturers; that those could not succeed unless they obtained first-rate articles to operate with. A long time ago, he continued, Mr. Bull in New York, made foil of a superior richness in color, and afterwards Mr. Abbey also. It was thought this fine color was owing to the purity of the metal; and how it was obtained remained a mystery for a number of years. Mr. L. and his brother after many experiments, discovered the peculiar hue was caused by heat, and that its color was changed by that agency. What dentists now so much desired—adhesiveness in foil—he had spent years in trying to get rid of. After foil has been annealed, the effect of annealing will pass off, and it can again be annealed by placing it over burning alcohol. (Mr. L., showed this by experiment; putting his foil on a small frame of common wire, that answered the purpose he said, as well as platinum plates or anything else.) Foil is adhesive in winter as in summer; but where there is moisture it will not adhere. He believed that foil would answer every purpose of crystal gold. By breaking the crystal gold one breaks the crystals, which are only of the size of the blocks. The bar gold has the same appearance as crystal gold. Manufacturers precipitated gold by proto-sulphate of iron (coperas), which mixed with the gold, and should be carefully washed off. It is, however, extremely difficult to separate all the iron from the gold; and it is the oxide of iron which by heat comes out upon the surface, and gives the foil its richness of hue. The oxide of iron insinuates itself through every particle of gold. Mr. Abbey's foil never adheres, unless he makes it so to do purposely. Crystal possesses no more adhesive quality than foil.

---

SECOND DAY—AFTERNOON SESSION.

*Question No. 2. Is the exalted sensibility of decayed teeth, the result of inflammation?*

Dr. Taft believed the determination of this question very important, for on an accurate knowledge of it depends the proper treatment of sensitive teeth. He believed their exalted sensibility did proceed from inflammation, although some of its signs, as redness and enlargement, can not, of course, be traced in osseous structures. The exalted sensibility decayed teeth have, is one of the most prominent indications of inflammation. Inflammation in the constitution generally induces inflammation in the teeth; and whenever there is a disposition to inflammation in the former, it manifests itself in the teeth. The treatment used for inflammation elsewhere may be employed satisfactorily for the teeth.

Dr. McCollum believed it was inflammation. The pulp of the tooth is excited, inflamed, when the dentine is sensitive.

Dr. Branch thought inflamed dentine a proper expression, and said it would yield in nine cases out of ten to a mild alkali. He had tried this method very successfully with many of his patients. Give persons complaining of their teeth a wash or powder, containing soda or some other gentle alkali; and it will seem to remove from the dentine the acid, the presence of which is an indication of inflammation in the bone.

Dr. Walker and Dr. Ulrey, were of the opinion it was inflammation, but could not easily give reasons for their belief. The question at best was one furnishing more scope for speculation than for scientific investigation.

Dr. James Taylor deemed the question of great importance. Inflammation had certain symptoms,—enlargement, redness, heat, pain. Here is a circulation in dentine, which is made up of small cells or tubes. No bodies but organized ones, those having nerves, can be inflamed. Dentine has pain and by analogy increased heat. There may be inflammation in dentine without exalted sensibility, as in cases preceding mortification. A particular form of disease is very prone to affect the teeth, and certain acids increase their sensibility. It was of the utmost importance to arrest disease in the mouth, and he believed the time would come when this great desideratum would be accomplished.

Dr. Osmond asked, if the exalted sensibility of decayed teeth resulted from inflammation, whether that inflammation was active or passive? The tooth of a healthy patient may have an active vitality and an active inflammation. Teeth must have circulation. In poor, scrofulous patients, there will be a passive inflammation. Stimulants affect sensitive dentine. Teeth, although looking well, decay suddenly sometimes. A body resists decay in proportion to its vitality. The sensibility of dentine is affected by reducing the vitality, and consequently the anti-phlogistic treatment must be good for active inflammation. Inflammation of dentine results from inflammation of the sheath of the nerves. General pathology, as understood and employed by the doctors of medicine, should regulate dentists in their treatment of sensitive dentine, rather than a disposition to use destructive agents.

Dr. Richardson expressed the opinion, that the exalted sensibility of decayed teeth in some cases depends upon inflammation, and in others, does not. In healthy patients the dentine may be in a state of exalted sensibility without inflammation. There is a great difference, as every one knows, in the fortitude of persons, owing to temperament and constitution. Exalted sensibility seems owing to the pulp. Dr. Salter, who had experimented considerably upon decayed teeth, had found in them a deposit in the pulp. Dr. Richardson had no doubt that inflammation often arises from the pulp; that the source of inflammation was frequently there. Exalted sensibility at the extremes of the nerves may exist independent of the dentine, in which he did not believe there was a vascular circulation. There can be no question about teeth being vitalized substances; they are as really so as anything else. Much of the common bone itself, however, is unvascular. He was of the opinion that dentine is capable of inflammation.

Dr. Spalding said, the high degree of sensibility of decayed teeth, is shown by the decomposition of dentine. May not the nerves or their sheaths be affected by their sensibility ?

Dr. Osmond said, that in the case of neuralgia, pressing upon a nerve always relieves; not so upon dentine, whose inflammation, as he said, arose from that of the sheath of the nerves. In perverted nutritions, decay of the teeth occurs more rapidly.

---

SECOND DAY—EVENING SESSION.

It had been determined in the afternoon session to depart from the discussion of the questions in numerical order, and to enter upon No. 6 and 7, considered together.

*What success has been obtained in fang filling? What is the best manner of filling the fangs of teeth?*

DR. BONSALL had had no great experience in fang filling. Some that he had filled eight or ten years before were still good. He had not much confidence in filling fangs, particularly those of molar teeth. He found difficulty in reaching the apex of a fang, in filling it. He did not think filling fangs remunerative either in time or money. He believed it better generally to take fangs out. Many of his patients, after having fangs filled, had come to him to have them extracted.

DR. BRANCH'S experience was precisely that of Dr. Bonsall. He had understood other dentists had been successful in fang filling, but he had not. He had known of one or two instances only of fang filling completely successful. His reason of failure, he believed, was owing to the non-removal of all the diseased or unhealthy matter in and about the fangs, and often to the fact that the nerves were not entirely killed.

DR. SPALDING said, that if there was anything in his profession upon which he prided himself, it was fang filling. His was Dr. Badger's method. It was essential in filling fangs that the entire nerve should be removed, so that the blood-vessels will contract upon themselves. A dentist should clean the fang perfectly, wipe out the cavity carefully, and operate immediately if the fang be healthy, the sooner the better. Dr. Spalding usually plugged fangs at the same sitting at which he removed the nerves. In preparing foil for the operation, by folding it of four thicknesses, into triangular pieces upon a broach; leaving a margin over, and twisting it firmly at the end. He gauged the diameter of the canal of the fangs, and selected a roll of foil of the same breadth as that diameter. He inserted a small piece of whalebone as far as he could, and then introduced his cylinders. He could in ordinary cases, fill a fang in five minutes. He deemed the success of fang filling as certain, almost as any other operation in his profession.

Some one having asked Dr. Spalding, what course he took when abscesses had formed; he replied, he could usually cure them, when there was neither sac nor absorption.

Dr. Spalding remarked in continuation, that he never set a pivot tooth without filling the fang. In such cases he took a small broach right angular at the termination; measuring the depth and breadth of the cavity, and fitted the gold to the measure. He had extracted some fangs that he had filled, and found their filling a complete success. He said many fangs were uselessly sacrificed. He often filled five or six fangs successfully in the same mouth. He removed the pulp with broaches, placing at the end a few fillets of cotton, entirely bleached, all parallel to the point. In filling fangs, his failures were not more than one in forty or fifty cases, and then mostly on account of previous abscesses. After filling fangs, he filled the pulp cavities with sulphate of lime, because they originally contained soft matter. He also capped nerves with plaster of paris. He often found nerves dead in fangs, although there were no traces of disease; the foramen had been closed by nature. Much caution should be observed in filling fangs for young persons, for their fangs often had hardly ossified.

Dr. Taft had pursued the same method as Dr. Spalding, for five or six years. He had often experienced difficulty in getting into the canal, and had constructed a drill to effect the purpose. He frequently made a hole through the centre of a tooth to the nerve-chamber and canal. It is not necessary to go beyond the foramen. He prepared his gold as Dr. Spalding had described. He thought it better after removing the nerve, to wait awhile to give time for cicatrization to take place. In young persons the foramen is large, and there was danger of putting in the gold too far. Different persons and different ages require different treatment. Inferior teeth were less easy to operate upon, because more dense; and it was more difficult to destroy their nerves.

Dr. Watt said his experience and success in filling fangs were about the same as Dr. Spalding and Dr. Taft's. In filling fangs where they were healthy, he removed the pulp. He washed out the canal with chloroform or creosote; then filled with gutta percha to exclude the atmosphere, and to retain the vapor of the remedy used. He relied on the closeing of the foramen by nature, and on the antiseptic power of remedies. When these had acted to his satisfaction, he washed out the canal with chloroform, dried with heated air, and filled the cavity of decay, leaving the canal unfilled.

Dr. McClelland had had such success in fang filling, that he regarded it as a crime to extract a tooth unless in case of abscess. He took particular pains to wash the cavity clean, and as soon as soreness subsided, he inserted his filling. In cases of abscess he had drawn off the pus and given relief. Filling a fang makes it sore usually, and patients are apt to bite with and handle it, which frequently inflames the fang. He advised them to the contrary. Camphor, chloroform and laudanum, will generally remove inflammation.

Dr. Stipher had filled some fangs successfully. In one he used a thread repeatedly as a test filling, but failed.

---

THIRD DAY—MORNING SESSION.

*No. 3.—What constitutional conditions will most predispose the teeth to decay?*

Dr. Richardson said, that the immediate cause of decay was the action of chemical agents upon the teeth. Certain conditions of the system, alter the character of the fluids of the mouth, so as to act upon the teeth. Dyspepsia or indigestion is the cause of acidity in the mouth, and that of decay. Of decay there may be a local and a constitutional cause, and the former may in time become the latter. Certain conditions of the system make constitutional changes. Derangements of the intestines affect the secretions of the mouth. Scrofula affects the salivary glands, influencing the mouth and generating acidity. All constitutional changes affect, and salivation perverts the secretions of the mouth. Mercury, he believed, had no direct action upon the teeth, only through the glands. Syphilis, in its secondary and tertiary stages, as well as other diseases, affected the secretions of the mouth, causing the teeth to decay.

Dr. James Taylor, believes that changes took place after the eruption of teeth through the gum. It was very necessary in the discussion of this question, to determine what changes in the constitution effect changes in the mouth. Certain temperaments are predisposed to disease, as was instanced in cases of cholera. The tooth, its character, its germ even, in his opinion, depended upon the temperament and constitution. Teeth are differently organized. The best organization—sanguine constitutions usually—had the best, the most complete teeth; and such constitutions resist the action of the disease. Proper and rich nourishment, and good, pure air, will remove, or at least diminish the lymphatic temperament — the lymphatic-sanguineous may become sanguino-lymphatic. Certain materials in the system have a tendency to keep the teeth sound and nourish them. In persons of a scorbutic temperament, bronchially affected, or tending to consumption, the teeth seem to grow softer instead of harder. Such instances had been observed in his own experience, and he had observed, after having checked the tendencies of changed secretions, that the teeth grew harder; showing the improvement of the system. In tracing the character of teeth, he had been able to learn of the general health of patients, and had informed their attendant physicians of their improvement, which information had proved well founded and correct. Dr. Taylor, referred in conclusion, to the great importance of this question, and said he only knew enough of it to make him extremely desirous of learning more.

Dr. Taft, was aware that teeth would decay while persons were ill, and after they had recovered. He had observed in his patients how their saliva had changed from alkaline to acid. Any reduction of the vital vigor will affect teeth, and dead ones decay more rapidly than living ones. Teeth decay in proportion to their vitality, to their reduction of resisting power. Mucus is naturally acid, but may be made more so. Where there is a disposition to inflammation of the mucus membrane, the mucus becomes more acid, and the teeth are more apt to decay. Where this disposition is, it also assists the decomposition of foreign substances about the teeth, influencing their decay. Regard should be paid to the question, whether the change in the saliva results from the blood or the salivary glands.

Dr. James Taylor believed the change in the saliva is owing particularly to the blood, from which this secretion is eliminated. A gland may be no more changed than the general system. The fluids that pass around the teeth are mucous secretions, no less than the saliva.

Dr. Spalding said, he had long been subject to bilious attacks, but his saliva never became acid until after his teeth had been injured by mercury. He would ask if certain medicines might not act directly upon the tissues of the system, independent of all action upon the secretions.

Dr. Osmond remarked, that people in this quarter of the globe, were more liable to decay of teeth than elsewhere; and the reason was, in his opinion, that they had vitiated systems and a remarkable deficiency of phosphate of lime therein. This may be ascribed to the manner of living here. It would be well to pay attention to this. Deficiency of phosphate of lime has a tendency to decay bones. In most

American constitutions, muriatic acid is produced in too great a quantity to be consumed by the lungs. Saleratus or alkalies affect the gastric juice. Large quantities of fat, sugar, etc., produce a super-abundance of lactic acid. Phosphate of lime hardens the teeth, and physicians sometimes prescribe it to pregnant mothers, that their children's teeth might be firm and sound. Such facts as these, if examined carefully, might show what influence the constitution has upon the teeth, and lead to some positive knowledge upon the subject that would be of incalculable advantage for the preservation of the teeth.

Dr. Branch believed that teeth decayed by chemical action, resulting necessarily from changes in the mouth. Our climate produced a high degree of nervous energy, and to this fact he attributed the rapid decay of teeth in America. He thought acidity of the mouth would influence the mucous membrane, instead of the mucous membrane causing acidity of the mouth. The mucus and saliva depend upon the manner in which the stomach manages its food. The Americans, as a people are more sensitive to the action of deleterious substances. He had known physicians to treat dyspepsia as a nervous disease successfully, and had also known that disease to result from the teeth. He himself had had such a case. The decay of the teeth generally, he thought owing to an over degree of nervous energy, instanced often in women when enciente.

Dr. Osmond said, that in healthy natures, carbon is eliminated and consumed. In less healthy natures, a large amount of lactic acid exists, which acid affects the mucous membrane.

Dr. James Taylor remarked, that the causes of disease are of two kinds, the predisposing and exciting. Chemical agents that act upon the teeth, are induced by constitutional changes. In all acute inflammation, we find an acid state of saliva. Mercury, in his opinion, hardly affected the dental organs, and when it did affect them, it was only through the secretions of the mouth. Years are required to remove the tendency to disease, in a certain part of the body; he did not think the teeth had as much power to resist disease as other parts of the body. In proportion, as we build up the vital power, we decrease the tendency to decay.

*Question No. 4. What has been the course pursued, and the success in the treatment of alveolar abscess?*

Dr. Bonsall generally removed the exciting cause in the treatment of alveolar abscess, and applied creosote to the part.

Dr. James Taylor said, that curing abscess without extracting the teeth would have been considered unprofessional some years since; but now to remove the teeth for such a purpose would be deemed so. The first step was to clean out the dental foramen and arrest the formation of matter. A discharge of matter through a diseased tooth is not an abscess, but may become so by stopping it. After operating upon the dental foramen, he applied creosote, chloride of zinc, alcohol, or camphor, and cotton again and again, until the foramen was perfectly clean. For a test plug he used patent thread. Cleaning out the fang is the main object; afterward remove all foreign matter; make a vacuum, and plug up firmly. He employed creosote as an antiseptic. When there was inflammation, generating a sac above the apex, he ran a fine instrument through the foramen, and let the pus escape; then used, in many instances, nitrate of silver, because it promoted healthy granulations. When the course of pus was tortuous, he ran a lance through the canal, making it straight and increasing its size; and in this way removed all the unhealthy matter. Abscesses can not always be treated through the canals of the fangs.

Dr. Knowlton had noticed in his practice the formation of a kind of gas in the bone of a tooth, which gives more pain than pus. The filling which he had removed from teeth was extremely offensive. He did not know how to reach or remove this gas, but had never used chloride of lime. One tooth which he had filled, he had been compelled afterwards to remove.

Dr. Branch had never attempted to operate upon an alveolar abscess, where there was a tendency to neuralgia. When he treated ulcerated fangs, he did it by external management; by external openings, he prevented the secretion of all morbid matter. In feeble patients with delicate constitutions he never endeavored to preserve dead fangs; as he believed it necessary in such cases to remove all irritating causes.

Dr. Spalding said, that for test plugs he had employed silk floss, and thread, and that he now used a wisp of cotton, not inserting it too tightly in the cavity, and changing it once in every twenty-four hours. By examining the cotton, by its color, odor, and general appearance, he determined when the fang was ready for filling. Scarcely a day passed on which he did not treat a case of alveolar abscess. He himself had had an ulcerated tooth for ten years, and from the beginning grew constantly worse, and yet never had been troubled by a neuralgic pang. A lower molar had troubled him, and he had tried every way to destroy the nerve, but ineffectually; and recently he had experienced a neuralgic pain either above the right or left eye.

Although the question was not disposed of, it had been determined the convention should adjourn this morning, and the discussion close at the hour of noon; and as it was considerably after that time when Dr. Spalding took his seat, all further consideration of No. 4, was indefinitely postponed.

